# Aberrant iron homeostasis, oxidative fiber enrichment, and activation of ketogenesis in muscle tissue of ISCU Myopathy patients

**DOI:** 10.1186/1753-6561-6-S3-P58

**Published:** 2012-06-01

**Authors:** Daniel R Crooks, Thanemozhi G Natarajan, Churning Chen, Hongzhan Huang, Manik C Ghosh, Wing-Hang Tong, Ronald G Haller, Cathy Wu, Tracey A Rouault

**Affiliations:** 1 Protein Information Resource, Department of Biochemistry, Molecular and Cellular Biology, Georgetown University Medical Center, Washington, DC 20057, USA; 2 Molecular Medicine Program, Eunice Kennedy Shriver National Institute of Child Health and Human Development, Bethesda, MD 20892, USA; 3 Center for Bioinformatics and Computational Biology, University of Delaware, Newark, DE 19711, USA; 4 Department of Neurology, University of Texas Southwestern Medical Center and VA North Texas Medical Center, and Neuromuscular Center, Institute for Exercise and Environmental Medicine, Dallas, Texas 75231, USA; 5

## 

ISCU Myopathy, a disease characterized by life-long exercise intolerance and impaired mitochondrial oxidative metabolism, is caused by deficiency of the Fe-S cluster scaffold protein ISCU. We performed gene expression analysis on muscle biopsies from ISCU Myopathy patients to elucidate which molecular processes were transcriptionally remodeled in response to impaired Fe-S cluster assembly. We found that ISCU depletion led to increased expression of the mitochondrial iron importer MFRN2 and the rate-limiting heme biosynthetic enzyme ALAS1. Gene expression and histologic studies demonstrated that patient muscle composition was shifted towards fewer glycolytic muscle fibers, more oxidative fibers, and increased capillary abundance. Paradoxically, mitochondrial fatty acid uptake and oxidation genes were coordinately up-regulated in patient muscles despite dramatic impairments of aconitase and succinate dehydrogenase activities. The ketogenic enzymes HMGCS2 and BDH1 were also significantly up-regulated, as was the secreted starvation response hormone, FGF-21. We propose that ketogenesis may be initiated to restore free coenzyme A levels and shunt fatty acid oxidation products to distal respiration-competent tissues when TCA cycle and/or respiratory chain function is sufficiently impaired in affected patient muscle fibers. Moreover, our work shows that plasma FGF21 is a sensitive non-invasive biomarker of ISCU Myopathy, in addition to other mitochondrial myopathies.

